# Employees Working from Home: Do Leadership Factors Influence Work-Related Stress and Musculoskeletal Pain?

**DOI:** 10.3390/ijerph20043046

**Published:** 2023-02-09

**Authors:** Jodi Oakman, Katrina A. Lambert, Victoria P. Weale, Rwth Stuckey, Melissa Graham

**Affiliations:** 1 Centre for Ergonomics and Human Factors, School of Psychology and Public Health, La Trobe University, Melbourne, VIC 3083, Australia; 2 School of Psychology and Public Health, La Trobe University, Melbourne, VIC 3083, Australia

**Keywords:** stress, musculoskeletal pain, leadership, psychosocial, working from home, risk factors

## Abstract

In March 2020, the COVID-19 pandemic necessitated a rapid public health response, which included mandatory working from home (WFH) for many employees. However, given the rapid change from traditional ways of working, evidence is limited on the role of leaders, managers, and supervisors in supporting their employees’ physical and mental health whilst WFH. The study aimed to examine the impact of leaders through their management of psychosocial working conditions on employees’ stress and musculoskeletal pain (MSP) levels whilst WFH. Methods: Data from 965 participants (230 males, 729 females, 6 other) involved in the Employees Working from Home (EWFH) study, collected in October 2020, and April and November 2021, were analysed. Generalised mixed-effect models were used to test relationships between psychosocial leadership factors and employees’ stress and MSP levels. Results: Higher quantitative demands are associated with increased stress (B: 0.289, 95%CI 0.245, 0.333), presence of MSP (OR: 2.397, 95%CI 1.809, 3.177), and increased MSP levels (RR: 1.09, 95%CI 1.04, 1.14). Higher levels of vertical trust decreased stress (B: −0.094, 95%CI −0.135, −0.052) and presence of MSP (OR: 0.729, 95%CI 0.557, 0.954). Role clarity decreased stress (B: −0.055, 95%CI −0.104, −0.007) and levels of MSP (RR: 0.93, 95%CI 0.89, 0.96). Working with interruptions was associated with increased stress (B: 0.199, 95%CI 0.119, 0.280) and MSP (OR: 1.834, 95%CI 1.094, 3.072). Conclusion: Leaders will need to take a broad view of job design, taking into account physical and psychosocial aspects of work, to effectively support employees WFH and manage stress and MSP.

## 1. Introduction

Prior to March 2020, working from home (WFH) was usually considered an employee benefit to improve the work–life interface, and was typically negotiated by individuals with their employers. However, in March 2020, the World Health Organization (WHO) declared a global pandemic due to COVID-19, and working lives across the globe were disrupted on an unprecedented scale [1]. Many countries instigated mandatory lockdowns to reduce the spread of the virus, which resulted in millions of workers shifting abruptly to WFH [2]. The sudden shift to WFH created challenges for organisations, which could not have predicted the rapid shift to home as the principal work environment [3]. In addition, the extended time period that employees were required to undertake mandatory WFH was not foreseeable. For example, in Melbourne, Australia, the population was in lockdown for a total of 262 days, one of the longest in the world [4]. The pandemic is ongoing, but vaccines and improved treatments have resulted in many countries learning to ‘live with COVID’, and the use of mandatory lockdowns has diminished [5]. However, the long periods of WFH have significantly influenced employee and employers’ expectations of working arrangements [6,7], in particular the location of work, which requires consideration of a range of factors, including leadership to ensure working conditions are appropriate [8]. Hybrid working models, a mix of WFH and office-based work, were once referred to as the model of the future [9]. However, the future has now arrived and to support the development of sustainable models of work, including hybrid options, more knowledge is required to understand the role of leadership in employees’ physical and mental health and the creation of high-quality working conditions, regardless of the location.

The Impact of the psychosocial wo”k en’ironment on employee health outcomes, including cardiovascular disease [10], mental health disorders [11,12], and musculoskeletal disorders [13,14,15], is well established and supported by an extensive evidence base. A number of theoretical models describe the relationships between various psychosocial factors, including the demand–control–support [16], effort–reward imbalance model [17], and job demands–resources model [18]. A long list of work-related psychosocial hazards has been identified, highlighting the complexity of organisational working environments and the broad range of potential contributing factors that impact employees’ mental and physical health. However, this complexity has resulted in challenges for workplaces to identify and then manage their psychosocial working conditions to ensure they are able to provide high-quality work environments to support their employees and mitigate negative health consequences [19]. To facilitate the measurement of psychosocial working conditions, the Copenhagen Psychosocial Questionnaire (COPSOQ) was developed [20]. The COPSOQ covers a broad range of psychosocial hazards and draws on the previously mentioned theoretical models to support workplaces and researchers in identifying adverse working conditions. Over 400 research papers have reported on the use of the COPSOQ [21], across many countries, and its broad coverage was the basis for its use in the current study.

Leaders, managers, and supervisors in organisations are integral to the creation of psychosocial working conditions, but are also uniquely placed to buffer the impacts on their employees [22]. A central component of a leader’s role is their influence on job design, defined as tasks or activities that employees complete for their organisations on a daily basis [23]. A cross-sectional study of Swedish baggage handlers found several leadership characteristics, measured using the COPSOQ, as well as recognition and support from supervisors and work organisation, were associated with self-reported low back pain [24]. Another Iranian study examined several psychosocial factors and the prevalence of musculoskeletal pain, finding that leadership was associated with reporting of low back pain [25]. 

Job design has an extensive history of research [26], with early research on job design focused on simplification and standardisation of tasks and a key focus on the removal of employee discretion or autonomy and control [23]. More recent research on job design has examined particular dimensions of jobs that influence employees’ mental and physical health, proposing the need for a more human-centred approach [26], which optimises rather than reduces key job characteristics. However, the COVID-19 pandemic has resulted in significant changes to working conditions and the location of work, exposing potential gaps in contemporary models of job design with subsequent impacts on employees’ health, which require further investigation to understand these psychosocial characteristics, which are within the remit of leaders and their employees’ physical and mental health.

A recent rapid review identified three key organisational characteristics, which influence employees’ mental and physical health whilst WFH, to support the optimisation of working conditions: job clarity, clear performance measures, and setting of appropriate workloads [27]. This review by Oakman and colleagues, and most other research on the psychosocial working environment, has been undertaken in traditional workplace settings where leaders and employees are collocated; it is unclear how this earlier research translates to more dispersed contemporary models of work. Training and resources to support leaders in their roles have been based largely on the premise of collocation; whether this remains appropriate is not yet clear. Exploration of the impact of leadership on employees’ health is important, and urgently required, to facilitate the development of evidence-informed guidance to assist leaders in effectively managing their employees and preventing adverse health outcomes. Furthermore, a longitudinal analysis is needed to provide a more in-depth exploration of the relationships between the environment and employees’ health. Based on previous research, it is hypothesised that psychosocial characteristics, which are within the remit of leaders, will influence employees’ work-related stress and MSP. To address this research gap, the current study aimed to explore the role of leadership through examining the impact of psychosocial working conditions on employees’ stress and musculoskeletal pain (MSP) levels whilst WFH. 

## 2. Materials and Methods

This study used data collected from the Employees Working from Home (EWFH) study conducted in Australia during the COVID-19 pandemic from October 2020 to November 2021. Sampling, recruitment, and a full description of the study profile for the EWFH study have been described elsewhere [28]. Briefly, convenience sampling was used to recruit a sample of Australian adults aged 18 or more years, who worked from home two or more days per week during the COVID-19 pandemic. Recruitment occurred via Facebook’s paid service, professional and personal networks, the La Trobe University Facebook page, and LinkedIn. 

**Figure 1:**
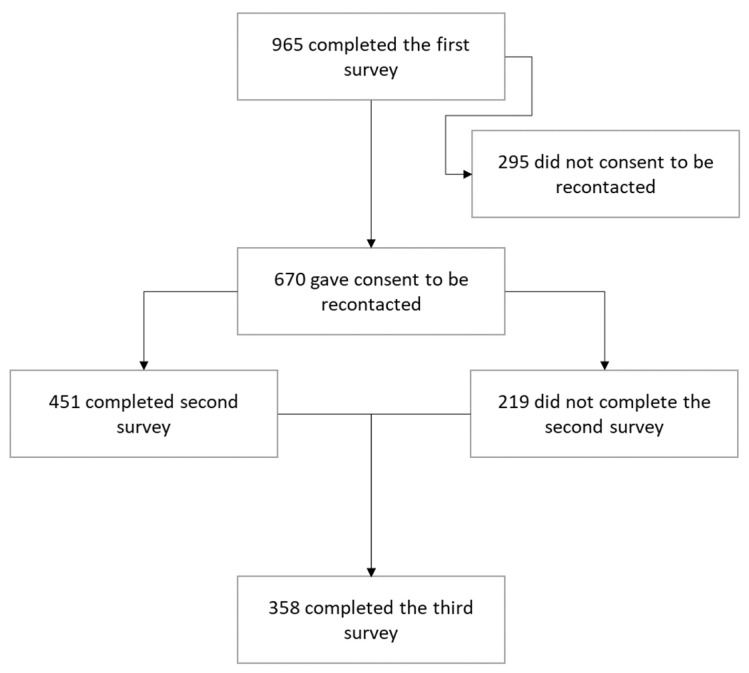
Flow of participants through the EWFH study.

Data were collected by questionnaire at three time points via Qualtrics XM software (Qualtrics, Provo, UT). All participants who consented to be recontacted after the first questionnaire were invited to participate in the second and third waves. Following the first questionnaire, nonresponders were provided with three reminders via email. Response rates for Waves 2 and 3 were 67% and 53%, respectively. The study flow is outlined in Figure 1.

Ethics approval was obtained through the La Trobe University Human Ethics Research Committee, approval number HEC20388. All study participants were provided with written information about the study and provided informed consent prior to participation.

### 2.1. Measures 

#### 2.1.1. Outcome Measures

##### Stress

Stress was measured using items from the COPSOQ [20]. Thirteen items were scored on a five-point Likert scale ranging from not at all (1) to all the time (5). An example item was “how often have you felt worn out?”. The 13 items (Cronbach alpha 0.939 [95%CI: 0.93, 0.95]) were averaged to form a single score of overall stress levels. 

##### Musculoskeletal Pain

Participants were asked to respond to the following question, “In the last 6 months, have you ever experienced discomfort or pain in part of your body, especially towards the end of your working day or night?”, and the response was yes or no. For those who responded yes, frequency and severity ratings of MSP were recorded separately for five body regions (neck/shoulders, hands/fingers, arms, middle to lower back, and hips/bottom/legs and feet) using a previously reported measure [29]. Response options for pain/discomfort frequency ranged from never (0) to almost always (4). Severity, if applicable, was scored using a three-point scale from mild (1) to severe (3). For those with pain, a pain score was derived by multiplication of frequency by severity for each body region and adding the resulting scores, creating a scale from 1 to 60.

### 2.2. Exposure Variables

#### 2.2.1. Psychosocial Work Environment

Sixteen questions were utilised from the COPSOQ, representing leadership: quantitative demands (4 items); social support from manager (2 items); vertical trust (3 items); quality of leadership (2 items); role clarity (3 items); and recognition (2 items). Responses were scored on a five-point scale, ranging from never/hardly ever (1) to always (5) with an option for not applicable to capture those participants who did not have a manager/supervisor or leader.

#### 2.2.2. Working from Home Environment

Location of the workspace was based on the item “When you are working at home, where do you usually work?”. Three response options were provided and coded as follows: Wherever—“I just find a place somewhere that’s free, such as on the kitchen table or other place”; Separate—“I have my own place in a separate room by myself”; and Interruptions—“I have my own place but in a room that can be busy with other people”. 

### 2.3. Covariates 

Age was based on the item “What is your age group?” 18–25 years; 26–35 years; 36–45 years; 46–55 years; 56 years and over. Categories were collapsed to 18–35 years; 36–45 years; 46–55 years; 56 years and over. Gender was based on the item “Are you: Male, Female, Other”, and the six participants who identified as Other were excluded from further analysis. 

Data were also collected on participants’ industry sector, role, business size, and domestic arrangement, including number and life stage of children.

### 2.4. Statistical Analyses

Demographic differences between participants who entered at least one follow-up questionnaire, those who did not consent to follow-up, and those who were lost to follow-up were calculated using the chi-squared test of independence. 

A generalised mixed-effect model with a Gaussian link function and random slope ID was used to model the relationships between psychosocial factors and stress. The presence of MSP was similarly modelled using the generalised mixed-effect model, binomial link, and random ID. Odds ratios (Ors) were calculated to facilitate the interpretation of results. The overall pain scores were modelled with a log link and negative binomial distribution to allow for the estimation of underdispersion or overdispersion. Estimation of dispersion avoids reliance on an assumption that the mean and variance of the outcome are equal. Rate ratios (RRs) were calculated to facilitate interpretation. The RR represents the change in the pain score in terms of percentage per unit increase in continuous independent variables. Multivariate model selection was conducted using the Akaike information criterion (AIC). Given the high correlation between measures of psychosocial demands (Supplementary Figure S1), potential multicollinearity was tested in all models using variation inflation factors.

Analysis was carried out in R version 4.1.1 “Kick Things” [30]. All tests of statistical significance were two-tailed, and *p* < 0 .05 was considered significant. 

## 3. Results

A total of 965 participants who completed the baseline survey were extracted for analysis (230 males, 729 females, 6 other). Between-group differences in follow-up status were identified between industry (*p* = 0.001) and sector (*p* = 0.018), although there was no significant difference in other demographic variables (Table 1). The majority of participants resided in Victoria (>80%), classed their role as ‘Professional’, and did not have children at home. 

All six psychosocial factors included in the analysis showed small but significant decreases in employee stress in univariate models, after controlling for age and gender (Table S1). The multivariate model with the lowest AIC (Table 2), that is, the most favourable model, showed no indications of multicollinearity. For each unit increase in quantitative demands, employee stress levels increased by 0.289 (95%CI: 0.245, 0.333). Conversely, employee stress levels decreased as social support from managers increased, with a similar pattern observed for vertical trust and role clarity. In relation to the location of work, experiencing interruptions resulted in an increase in employee stress levels compared to those with a separate space. Other factors related to higher stress levels included being female, whilst increased age was associated with reduced stress levels. The initial follow-up was associated with an overall decrease in stress among the participants; however, by the second follow-up, on average stress levels had returned to baseline. 

The presence of MSP was associated with increased quantitative demands and vertical trust in both univariate (Table S2) and the best-fitting multivariate model (i.e., lowest AIC), as shown in Table 3. Working in a room with interruptions and being female were also associated with having MSP (OR: 1.834 95%CI: 1.09, 3.07 and OR: 3.09 95%CI: 1.80, 5.29, respectively) in the multivariate model.

For those participants who reported having MSP, further analysis was undertaken. All six psychosocial measures showed small but significant decreases in employee MSP levels in univariate models (Table S3). The multivariate model with the lowest AIC (Table 4) retained role clarity, with a 7% decrease in the rate ratio of pain for every unit increase in role clarity (RR: 0.93 95%CI: 0.89, 0.96). The modelling also showed a 9% increase in pain for every unit increase in quantitative demands. The rate ratio for working ‘Wherever’ was 1.13 (95%CI: 1.02, 1.24) times the rate for working in a separate room; likewise, the rate for females is 1.29 (95%CI: 1.14, 1.46) times that of males and those 46–55 years and 56 years and over have significantly higher RRs than the reference group (18–35 years), holding the other variables at constant.

## 4. Discussion

In the context of significant change to working lives due to the impact of the COVID-19 pandemic, the aim of the current study was to examine the influence of leaders, through their role in managing the psychosocial working conditions, on employees’ stress and MSP levels. Specifically, psychosocial factors identified as relevant in pre-pandemic work environments to employees’ physical and mental health, and within the potential remit of leaders, were selected for analysis in the study. Findings from the current study illustrate high quantitative demands are associated with increased stress, having MSP, and also MSP of greater severity. Higher levels of support, vertical trust, and role clarity were associated with reduced employee stress, whilst high vertical trust was associated with reduced MSP presence, and high role clarity was associated with reduced levels of MSP. These results support the proposed hypothesis that those psychosocial characteristics, which are within the remit of leaders, will influence employees’ work-related stress and MSP. In relation to the location of work within the home environment, employees unable to find a location to work without interruptions reported higher stress and MSP levels than those who had access to a separate room. The current study makes an important contribution to the literature on WFH, as very few studies have examined the specific impacts of psychosocial working conditions on employees’ stress and MSP levels during mandatory WFH.

The context in which the data were collected is important and a key strength of the study. Three periods of data were collected from employees during the COVID-19 pandemic, many experiencing one of the longest lockdowns in the world. As such, the study affords unique insights into the role of psychosocial working conditions on employees’ health whilst WFH. Through examining the impact of the psychosocial work environment on stress and MSP separately, we gain clear insights into the pattern of exposures on both outcomes and, importantly, the degree of overlap in these exposures. The degree to which the psychosocial work conditions influence stress and MSP in the context of WFH is a significant contribution to the literature and to the knowledge base for practitioners in occupational health.

Workplaces typically take a siloed approach to managing mental and physical health, with separate policies and procedures for each [31,32]. Typically, mental health is managed by human resources personnel, and musculoskeletal and other health disorders are managed by OHS. This siloed approach was prevalent pre-COVID-19, and is a significant barrier to improving psychosocial working conditions and work design in workplaces, particularly in organisations where MSDs were perceived to be the main problem [33]. The need for a comprehensive approach to the management of employees’ health, which takes into account all aspects of the work environment, has been previously proposed [34], but the translation of this research into standard workplace risk management approaches is limited. Distributed models of work require new ways of thinking, as supported by the findings from the current study, which demonstrates the pivotal role of psychosocial work conditions in the development of employee stress and MSP.

The findings from this study provide insights into the importance of job design, which accounts for not only the quantity of work required by an employee, but also a much broader range of factors, including social support, vertical trust, and role clarity. Through adopting principles of good job design [35], leaders within organisations can address these significant contextual factors, identified in the current study, to positively impact employees’ health (mental and physical). However, a key consideration is the position of leaders and ensuring they are appropriately skilled in understanding the important role they have in job design and the psychosocial working conditions that ensue. The key role of leaders in job design has been proposed before, but leaders’ knowledge of what psychosocial working conditions are, and how they should be managed, has been identified as a barrier to effective risk management [32,36].

Work undertaken in the home has not been included in traditional pre-pandemic workstation assessment. Typically, workstation assessments have focused on the physical attributes, including the desk, chair, computer height, and whether separate peripherals are available such as a keyboard and mouse [37]. Findings from the current study showed that workstation location was important for both stress and MSP. Not surprisingly, working in a space with interruptions was predictive of increased stress levels and having MSP. For those employees with MSP, pain was increased when they did not have access to a dedicated workstation. Employees who have MSP are likely to need a tailored workspace, with appropriate equipment to assist with managing their condition [38], and may require support from their employers to optimise their workspace away from the traditional office. Support may include equipment but also modifications to job design to enable flexibility, so employees can tailor their work to their capacity, which is particularly important in conditions where symptoms are variable.

As we continue living and working during the COVID-19 pandemic, organisations need to ensure they are providing adequate resources and support to their employees. The findings from this study provide clear support for adopting a comprehensive approach that ensures high-quality psychosocial working conditions are provided to support employees’ mental and physical health. Leaders, responsible for managing people working both in the office and at home, may require additional support to ensure they are informed about their role in this new WFH and hybrid working context, and how to best support their employees. In a hybrid working model, only ensuring that employees have the adequate physical resources to undertake their role will be insufficient. High-quality psychosocial working conditions have been shown to be critical to ensure workers can work effectively, without negative impacts on their mental and physical health.

### Limitations

A strength of the study was the prospective design with the three data collection waves over an 18-month period during the COVID-19 pandemic. The study has a high proportion of participants from Victoria, Australia, the city that experienced the most days in lockdown than many other cities across the world. The EWFH study population is a convenience sample, based in Australia, which may restrict the generalisability of the results. The sample contains a higher proportion of women compared to men. As with all prospective studies, the dropout rates of participants are a further limitation. All measures are based on self-report; objective measures were not practicable during the pandemic situation in which these data were collected. Data were not collected on participants’ MSP or stress levels prior to the COVID-19 pandemic. The question regarding workstation location was developed for the purpose of the current study as no suitable published item was identified. The perspectives are those of employees, not their leaders, an important contribution. Finally, the data for the present study were collected during the COVID-19 pandemic; as such, the broader environmental context involved significant changes to many aspects of lives beyond work and is an important factor in the interpretation of the study results. 

## 5. Conclusions

The findings from this study support the important role of leadership, as measured by several psychosocial factors (quantitative demands, social support from managers, vertical trust, quality of leadership, and role clarity), and the influence on employees’ mental and physical health when working from home. Potential limitations in knowledge and skills to manage psychosocial working conditions mean that traditionally, a key leadership focus has been on managing the physical workstation for employees WFH, rather than considering the broader job design and how they can optimise this in a remote setting. Findings from this study provide evidence for the major role of several psychosocial factors in the development of stress and MSP, which may potentially be addressed through improved work design; it is important that those in leadership roles are provided with support to effectively manage the psychosocial working conditions, and thus optimise the mental and physical health of employees WFH.

## Figures and Tables

**Table 1:** Demographic characteristics of baseline participants characteristics according to follow-up status.

	All (N = 965)	Did Not Consent to Follow-Up (n = 293)	Lost to Follow-Up (n = 159)	Entered Follow-Up (n = 513)	*p*-Value ^a^
**Age**					0.630
18–35 years	210 (26.58%)	31 (26.05%)	51 (32.08%)	128 (25.00%)	
36–45 years	243 (30.76%)	36 (30.25%)	48 (30.19%)	159 (31.05%)	
46–55 years	207 (26.20%)	29 (24.37%)	37 (23.27%)	141 (27.54%)	
56 years and over	130 (16.46%)	23 (19.33%)	23 (14.47%)	84 (16.41%)	
**State**					0.172
Victoria	808 (83.73%)	237 (80.89%)	131 (82.39%)	440 (85.77%)	
Other	157 (16.27%)	56 (19.11%)	28 (17.61%)	73 (14.23%)	
**Industry**					0.001
Education and Training	322 (33.37%)	101 (34.47%)	41 (25.79%)	180 (35.09%)	
Financial and Insurance Services	49 (5.08%)	21 (7.17%)	17 (10.69%)	11 (2.14%)	
Healthcare and Social Assistance	138 (14.30%)	29 (9.90%)	25 (15.72%)	84 (16.37%)	
Information, Media and Telecommunications	45 (4.66%)	17 (5.80%)	7 (4.40%)	21 (4.09%)	
Professional, Scientific, and Technical Services	207 (21.45%)	61 (20.82%)	33 (20.75%)	113 (22.03%)	
Public Administration and Safety	98 (10.16%)	30 (10.24%)	14 (8.81%)	54 (10.53%)	
Transport, Postal and Warehousing	32 (3.32%)	9 (3.07%)	12 (7.55%)	11 (2.14%)	
Other	74 (7.67%)	25 (8.53%)	10 (6.29%)	39 (7.60%)	
**Sector**					0.018
Public sector	524 (54.30%)	150 (51.19%)	74 (46.54%)	300 (58.48%)	
Private sector	288 (29.84%)	104 (35.49%)	54 (33.96%)	130 (25.34%)	
Not-for-profit sector	120 (12.44%)	28 (9.56%)	25 (15.72%)	67 (13.06%)	
Self-employed	33 (3.42%)	11 (3.75%)	6 (3.77%)	16 (3.12%)	
**Role**					*
Manager	157 (16.27%)	42 (14.33%)	33 (20.75%)	82 (15.98%)	
Professional	587 (60.83%)	185 (63.14%)	89 (55.97%)	313 (61.01%)	
Clerical or Administrative Workers	199 (20.62%)	59 (20.14%)	31 (19.50%)	109 (21.25%)	
Community & Personal Service Worker	10 (1.04%)	0 (0.00%)	4 (2.52%)	6 (1.17%)	
Sales Worker	9 (0.93%)	5 (1.71%)	2 (1.26%)	2 (0.39%)	
Techs, Trade, Machine Operators & Drivers	3 (0.31%)	2 (0.68%)	0 (0.00%)	1 (0.19%)	
**Business Size**					*
Sole Trader	29 (3.01%)	11 (3.75%)	2 (1.26%)	16 (3.12%)	
Small Business	74 (7.67%)	20 (6.83%)	15 (9.43%)	39 (7.60%)	
Medium business	95 (9.84%)	38 (12.97%)	17 (10.69%)	40 (7.80%)	
Large business	767 (79.48%)	224 (76.45%)	125 (78.62%)	418 (81.48%)	
**Domestic Arrangements**					0.678
Single person household	124 (12.85%)	39 (13.31%)	23 (14.47%)	62 (12.09%)	
Adults only	418 (43.32%)	118 (40.27%)	68 (42.77%)	232 (45.22%)	
Dependents	423 (43.83%)	136 (46.42%)	68 (42.77%)	219 (42.69%)	
**Number of Children**					0.701
None	623 (64.56%)	188 (64.16%)	105 (66.04%)	330 (64.33%)	
1	119 (12.33%)	44 (15.02%)	18 (11.32%)	57 (11.11%)	
2	181 (18.76%)	49 (16.72%)	30 (18.87%)	102 (19.88%)	
3 or more	42 (4.35%)	12 (4.10%)	6 (3.77%)	24 (4.68%)	
**Child’s Life stage ^b^**					
Pre-school	94 (27.49%)	11 (10.38%)	5 (9.26%)	11 (5.98%)	0.612
Grades Prep-2	90 (26.32%)	21 (20.00%)	13 (24.07%)	33 (18.03%)	0.751
Grades 3–6	111 (32.46%)	25 (23.81%)	14 (25.93%)	51 (27.87%)	0.961
Grades 7–10	104 (30.41%)	33 (31.43%)	18 (33.33%)	60 (32.79%)	0.722
Grades 11–12	56 (16.37%)	29 (27.62%)	18 (33.33%)	57 (31.15%)	0.781

^a^. Chi-squared test of independence between follow-up status. * Chi-square not presented due to small expected values. ^b^. Multiple answers: percentages may not equal 100%.

**Table 2:** Associations between stress and psychosocial factors and workspace.

	Estimate (95%CI)	GVIF
Psychosocial *		
Quantitative demands	0.289 (0.245, 0.333)	1.05
Social support from managers	−0.056 (−0.099, −0.014)	1.37
Vertical trust	−0.094 (−0.135, −0.052)	1.24
Quality of leadership	0.011 (−0.029, 0.052)	1.47
Role Clarity	−0.055 (−0.104, −0.007)	1.20
**Location of Workspace**		1.01
Separate Room	Reference	
Interruptions	0.199 (0.119, 0.280)	
Wherever	0.067 (−0.036, 0.169)	
**Gender**		1.02
Male	Reference	
Female	0.233 (0.135, 0.330)	
**Age**		1.01
18–35 years	Reference	
36–45 years	−0.226 (−0.326, −0.126)	
46–55 years	−0.327 (−0.435, −0.218)	
56 years and over	−0.415 (−0.546, −0.284)	
**Questionnaire**		1.01
Baseline	Reference	
One	−0.237 (−0.299, −0.176)	
Two	−0.015 (−0.079, 0.048)	

* Based on measures ranked 1 to 5. GVIF= generalised variation inflation factor.

**Table 3:** Associations between musculoskeletal pain and psychosocial factors and workspace.

	Odds Ratio (95%CI)	GVIF
Psychosocial *		
Quantitative demands	2.397 (1.809, 3.177)	1.06
Social support from managers	0.949 (0.722, 1.248)	1.38
Vertical trust	0.729 (0.557, 0.954)	1.26
Quality of leadership	1.135 (0.882, 1.462)	1.44
Role clarity	1.033 (0.760, 1.404)	1.22
**Location of workspace**		1.01
Separate room	Reference	
Interruptions	1.834 (1.094, 3.072)	
Wherever	1.588 (0.820, 3.076)	
**Gender**		1.02
Male	Reference	
Female	3.089 (1.804, 5.290)	
**Age**		1.01
18–35 years	Reference	
36–45 years	0.864 (0.479, 1.560)	
46–55 years	0.568 (0.305, 1.059)	
56 years and over	0.724 (0.347, 1.512)	
**Questionnaire**		1.01
Baseline	Reference	
One	0.838 (0.550, 1.277)	
Two	1.546 (0.974, 2.455)	

* Based on measures ranked 1 to 5. GVIF= generalised variation inflation factor.

**Table 4:** Associations between musculoskeletal pain levels * and psychosocial factors and workspace.

	RR (95%CI)	GVIF
Psychosocial **		
Quantitative demands	1.09 (1.04, 1.14)	1.04
Role Clarity	0.93 (0.89, 0.96)	1.04
**Location of Workspace**		1.01
Separate Room	Reference	
Interruptions	1.07 (0.99, 1.16)	
Wherever	1.13 (1.02, 1.24)	
**Gender**		1.02
Male	Reference	
Female	1.29 (1.14, 1.46)	
**Age**		1.01
18–35 years	Reference	
36–45 years	1.01 (0.90, 1.13)	
46–55 years	1.17 (1.03, 1.32)	
56 years and over	1.20 (1.03, 1.40)	
**Survey time periods**		1.01
Baseline	Reference	
One	0.94 (0.89, 1.00)	
Two	0.95 (0.90, 1.01)	

* Only people with MSP included in analysis. ** Based on measures ranked 1 to 5. GVIF = generalised variation inflation factor.

## Data Availability

Data can be provided by request from the corresponding author, subject to ethical requirements.

## Supplementary Materials

The following supporting information can be downloaded at: https://www.mdpi.com/article/10.3390/ijerph20043046/s1, Figure S1: Correlation between measures of leadership; Table S1–S3: Stress analysis–univariate (adjusted gender and age); S2 Pain (presence) analysis–univariate (adjusted gender and age); S3: Total pain score analysis–univariate (adjusted gender and age).
