# Supplementary material for: Employees Working from Home: Do Leadership Factors Influence Work-Related Stress and Musculoskeletal Pain?
Source: Int J Environ Res Public Health. 2023 Feb 9;20(4):3046. doi: 10.3390/ijerph20043046 (PMC9964430; doi:10.3390/ijerph20043046)
Supplement: Supplementary file 1 [file ijerph-20-03046-s001.zip › ijerph-2197877-supplementary.pdf]

1 Supplementary Material

Figure S1: Correlation between measures of leadership

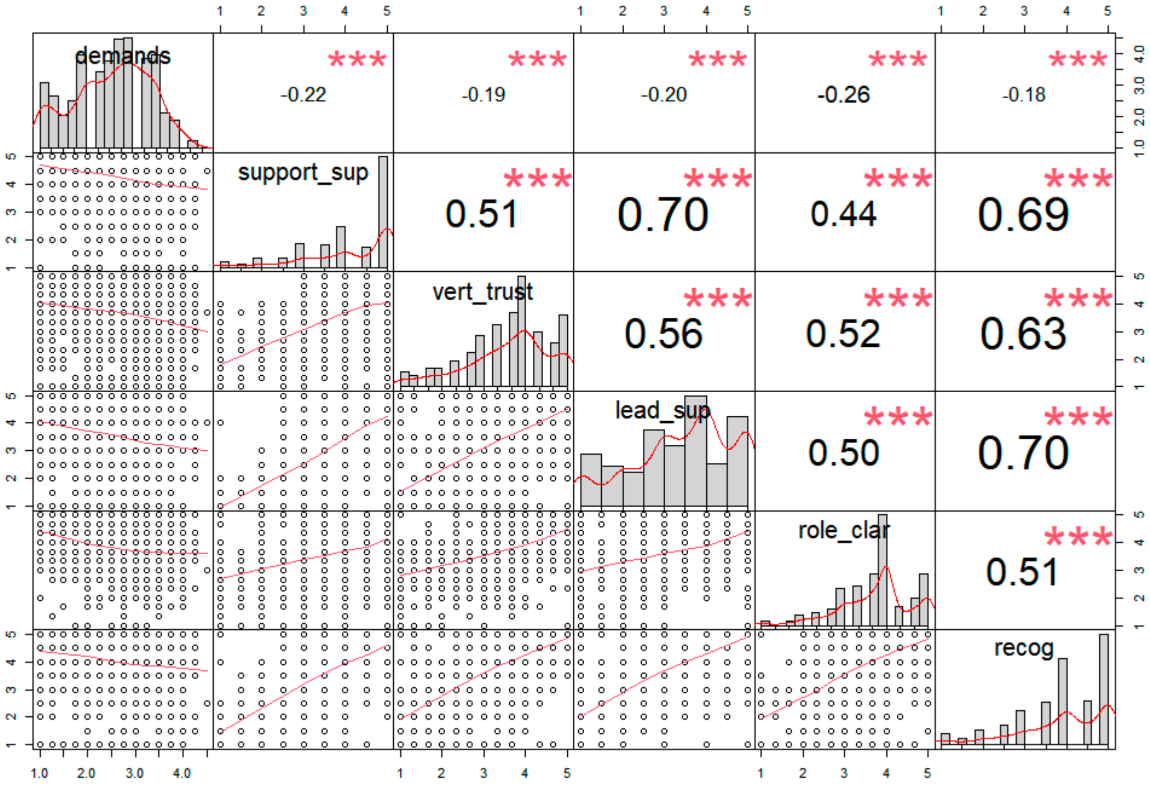

demands = Quantitative Demands; support_sup = Social support from managers; vert_trust = Vertical trust; lead_sup = Quality of leadership; role_clar = Role Clarity; recog = Recognition

Table S1: Stress analysis – univariate (adjusted gender and age)

	B (95%CI)
Survey	
Baseline	Reference
One	-0.162 (-0.216, -0.107)
Two	0.0246 (-0.035, 0.084)
Quantitative Demands	0.305 (0.264, 0.346)
Social support from managers	-0.139 (-0.172, -0.107)
Vertical trust	-0.154 (-0.188, -0.119)
Quality of leadership	-0.111 (-0.141, -0.080)
Role Clarity	-0.184 (-0.225, -0.143)
Recognition	-0.133 (-0.166, -0.100)
Location of Workspace	
Separate Room	Reference
Interruptions	0.224 (0.138, 0.309)
Wherever	0.132 (0.026, 0.239)

Table S2 Pain (presence) analysis – univariate (adjusted gender and age)

	OR (95%CI)
Survey	
Baseline	Reference
One	0.86 (0.59, 1.24)
Two	1.82 (1.18, 2.82)
Quantitative Demands	2.21 (1.70, 2.88)
Social support from managers	0.87 (0.71, 1.07)
Vertical trust	0.78 (0.63, 0.97)
Quality of leadership	0.88 (0.73, 1.06)
Role Clarity	0.73 (0.56, 0.95)
Recognition	0.91 (0.75, 1.12)
Location of Workspace	
Separate Room	Reference
Interruptions	1.95 (1.14, 3.33)
Wherever	1.54 (0.79, 2.98)

Table S3: Total pain score analysis – univariate (adjusted gender and age)

	IRR (95%CI)
Quantitative Demands	1.12 (1.07, 1.18)
Social support from managers	0.96 (0.93, 0.99)
Vertical trust	0.94 (0.90, 0.97)
Quality of leadership	0.95 (0.92, 0.99)
Role Clarity	0.89 (0.86, 0.93)
Recognition	0.95 (0.91, 0.98)
Location of Workspace	
Separate Room	Reference
Interruptions	1.09 (1.01, 1.18)
Wherever	1.13 (1.03, 1.24)
Survey	
Baseline	Reference
One	0.96 (0.92, 1.01)
Two	0.95 (0.91, 1.00)
